# Loss of CCR7 Expression on CD56^bright^ NK Cells Is Associated with a CD56^dim^CD16^+^ NK Cell-Like Phenotype and Correlates with HIV Viral Load

**DOI:** 10.1371/journal.pone.0044820

**Published:** 2012-09-19

**Authors:** Henoch S. Hong, Fareed Ahmad, Johanna M. Eberhard, Nupur Bhatnagar, Benjamin A. Bollmann, Phillip Keudel, Matthias Ballmaier, Margot Zielinska-Skowronek, Reinhold E. Schmidt, Dirk Meyer-Olson

**Affiliations:** 1 Klinik für Immunologie und Rheumatologie, Medizinische Hochschule Hannover, Hannover, Germany; 2 Division of Immunology, New England Primate Research Center, Harvard Medical School, Southborough, Massachusetts, United States of America; 3 Pädiatrische Hämatologie und Onkologie, Medizinische Hochschule Hannover, Hannover, Germany; University of Sydney, Australia

## Abstract

NK cells are pivotal sentinels of the innate immune system and distinct subpopulations in peripheral blood have been described. A number of studies addressed HIV-induced alterations of NK cell phenotype and functionality mainly focusing on CD56^dim^CD16^+^ and CD56^−^CD16^+^ NK cells. However, the impact of HIV-infection on CD56^bright^ NK cells is less well understood. Here we report a rise of CD56^bright^ NK cells in HIV-infected individuals, which lack CCR7-expression and strongly correlate with HIV viral load. CCR7^−^CD56^bright^ NK cells were characterized by increased cytolytic potential, higher activation states and a more differentiated phenotype. These cells thus acquired a number of features of CD56^dim^CD16^+^ NK cells. Furthermore, CD56^bright^ NK cells from HIV patients exhibited higher degranulation levels compared to uninfected individuals. Thus, chronic HIV-infection is associated with a phenotypic and functional shift of CD56^bright^ NK cells, which provides a novel aspect of HIV-associated pathogenesis within the NK cell compartment.

## Introduction

NK cells are effector cells of the innate immune system, which can spontaneously sense and lyse virus-infected cells [1], [2]. Distinct NK cell subpopulations have been described. The majority of human NK cells in peripheral blood are CD56^dim^CD16^+^ cells whereas CD56^bright^ cells only constitute approximately 10% of the blood NK cell pool [3]. Among other markers, CD56^bright^ NK cells are characterized by high expression of type II membrane glycoprotein CD94, L-selectin CD62L and lymph-node homing receptor CCR7 [4], [5] but low expression of the low affinity IgG-Fc-receptor III (CD16), killer cell immunoglobulin-like receptors (KIRs) and cytolytic molecules such as perforin and granzyme B, which are predominantly features of CD56^dim^CD16^+^NK cells [1]. Thus, NK cell subsets seem to have distinct roles in the immune response. Generally, CD56^dim^CD16^+^ NK cells are viewed as the cytotoxic NK cell subpopulation whereas CD56^bright^ NK cells were described to have regulatory functions by means of cytokine production, such as IFN-γ and TNF among others [1], [3].

Recent studies have emphasized the pivotal contributions of NK cells in the host defense against HIV [6], [7]. However, a number of defects in NK cell biology caused by HIV-infection have been documented [8]. We have shown an association of chronic HIV-infection with a significant decline of less differentiated and functionally more active CD56^dim^CD16^+^ NK cells, which are either CD57^−^ or CD57^dim^ [9]. In addition, we and others characterized an expansion of CD56^−^CD16^+^ NK cells in HIV infection with a terminally differentiated phenotype [10]–[12], which might reflect an increased turnover of NK cells in chronic HIV infection [13]. Nonetheless, little is known about the impact of HIV viremia and chronic HIV-1 infection on CD56^bright^ NK cells.

CD56^bright^ NK cells have been suggested to be less differentiated NK cells, which can give rise to CD56^dim^CD16^+^ NK cells [14] and an accumulating body of evidence seems to corroborate this view [5], [9], [15]–[19]. Enhanced cytolytic activity of these cells has been previously associated with HIV-infection [11], [20]. Here we show that high HIV-1 viral load significantly correlates with a loss of CD56^bright^ NK cells expressing CCR7. CCR7^−^CD56^bright^ NK cells exhibited a number of features resembling CD56^dim^CD16^+^ NK cells. These results thus present evidence for profound alterations of CD56^bright^ NK cells in HIV-infection.

## Materials and Methods

### Ethical Approval

The study was performed in strict accordance with the ethical principles as outlined in the WMA Declaration of Helsinki. All study subjects gave written, informed consent prior to their participation. The protocol was approved by the local ethics committee (Votum der Ethikkommission der MHH No. 3150).

### Study Subjects

We obtained peripheral blood samples from 37 untreated and 15 treated HIV-seropositive subjects on highly active antiretroviral therapy (HAART) and 16 uninfected individuals in the HIV outpatient clinic of the Medizinische Hochschule Hannover (MHH). A summary of the demographical data of the studied groups is shown in Table 1 and more detailed information on the HIV-seropositive blood donors are provided in Table S1. Plasma HIV-1 RNA levels were determined using the VERSANT-HIV-1 RNA Assay, version 3.0 (bDNA, Bayer Diagnostics, Berkeley, CA) and absolute lymphocyte counts were routinely determined by differential hemograms. Frequencies of CD4^+^ T cells and other lymphocyte subpopulations were determined by flow cytometry using a cocktail of diagnostic staining antibodies from Beckman Coulter either directed against CD45, CD3, CD4 and CD8 or CD45, CD56, CD19, CD3 and CD16. Absolute CD4^+^ T cell counts were calculated by determining their frequency of the total lymphocytes.

**Table 1:** Summary of demographical data of study participants.

Number ofsubjects	Group	Male/Femaleratio	Mean age ± SD	Mean CD4 T cell count(n/µl) ± SD	Median Viral load (n/ml)	Mean CD4/CD8 T cellratio ± SD
15	HIV^+^, ART	2.75	44±11	480±212	undetectable	0.66±0.28
38	HIV^+^, Untreated	2.08	41±14	467±271	11450	0.48±0.31
16	Control	1.67	38±13	848±191	seronegative	1.67±0.52

The profiles of all study participants are shown in a summary.

### Isolation of Mononuclear Cells

PBMCs were isolated from fresh blood as described previously [12], [21]. Aliquots of 10^7^ PBMCs each were cryopreserved in heat-inactivated FCS supplemented with 10% dimethyl sulfoxide (DMSO) (Merck).

### Phenotypic Analysis of NK Cells by Flow Cytometry

A list of monoclonal antibodies employed in this study is available upon request. Staining and flow cytometric analysis was performed as described before [9]. To define absolute numbers of NK cell subpopulations, we first determined the percentages of these subsets of total lymphocytes and then determined their absolute numbers using the absolute counts of lymphocytes. Intracellular expression of perforin, granzyme B and Ki-67 was analyzed in unstimulated NK cells using ‘Fix and Perm’ kit (Invitrogen) according to the instructions provided by the manufacturer. CD56^bright^ NK cells were only analyzed if at least 1,000 gated events were acquired.

### CD107a Degranulation Assay and Intracellular Cytokine Staining

Functional NK cell assays were performed as described previously [9]. Briefly, sorted NK cells or PBMCs were stimulated with 100 ng/ml IL-12, 10 ng/ml IL-15 and/or K562 cells at an E:T ratio of 2∶1 or 100,000 K562 cells per 1 million PBMCs. CD107a degranulation after 6 hours of stimulation and incubation was detected as described before [9], [22]. Anti-IFN-γ Pacific-Blue (clone 4S.B3, Biolegend) and anti-TNF Alexa Fluor 700 (clone MAb11, BD Biosciences) were used to detect intracellular expression of cytokines.

### NK Cell Differentiation Assay

CCR7^+^CD56^bright^ NK cells were sorted and suspended in RPMI 1640 supplemented with 10% FCS (Biochrom), 100 U/ml penicillin and 100 µg/ml streptomycin, 2 mmol/l l-glutamine and 1 mmol/l sodium pyruvate. The purity of sorted cells exceeded 95%. CCR7^+^CD56^bright^ NK cells were cultured at a cell density of 100,000 cells per well in the presence or absence of 100 U/ml IL-2, 25 ng/ml IL-12 and 25 ng/ml IL-15. At days 0, 3 and 5 phenotypic analyses were performed by flow cytometry.

### Statistical Analysis

GraphPad Prism (version 5.0) software was used for statistical evaluation of data. Pearson analysis was employed to determine correlations. Unpaired, two-tailed t test when comparing two groups or One-way ANOVA followed by Tukey test when comparing more than two groups were performed and *P* values of less than 0.05 were considered significant.

## Results

**Figure 1:**
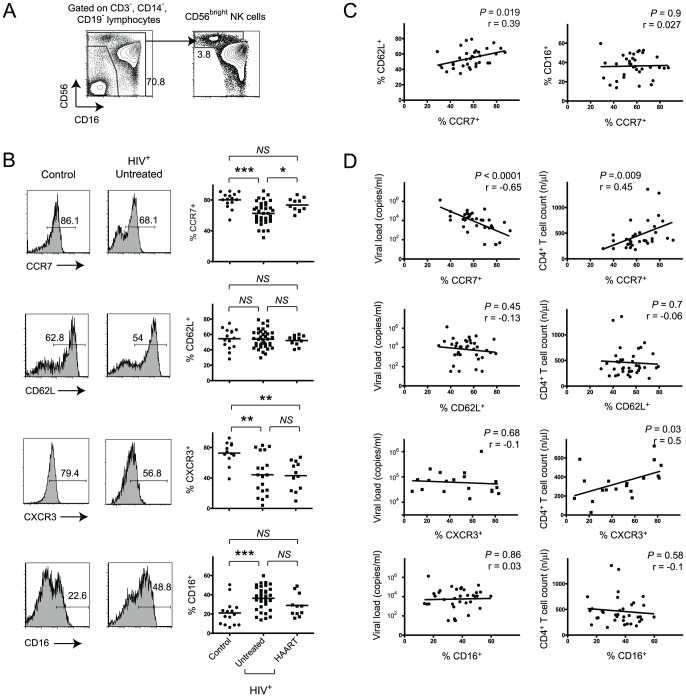
Loss of CCR7-expressing CD56^bright^ NK cells correlates with clinical disease markers. (A) Representative flow cytometry plots defining CD56^bright^ NK cells. Numbers indicate percentage of the gated population. (B) Representative CCR7, CD62L, CXCR3 and CD16 expression data and summary data all gated on CD56^bright^ NK cells. Horizontal bars in dot plot indicate mean values. (C) Pearson’s correlation analysis between frequencies of either CD62L^+^ or CD16^+^CD56bright NK cells and CCR7^+^CD56^bright^ NK cells in untreated HIV-seropositive patients. (D) Pearson’s correlation analysis between frequencies of CCR7^+^, CD62L^+^, CXCR3^+^ or CD16^+^ cells of total CD56^bright^ NK cells with either viral load or CD4^+^ T cell counts in untreated HIV-positive subjects. *, *P*<0.05; ***, *P*<0.001; *NS* – not significant.

We excluded T cells, B cells and monocytes from the analysis as previously described [9] and identified CD56^bright^ NK cells as shown in Fig. 1A. This gating strategy included CD16^+^ expressing CD56^bright^ NK cells, which were still distinguishable from CD56^dim^CD16^+^ NK cells due to their bright CD56 expression. We first assessed the expression of CCR7, CD62L, CXCR3 and CD16 on CD56^bright^ NK cells in HIV-seronegative donors as well as in a cohort of HIV-patients, which included treated and untreated subjects. There was a substantial decrease of CCR7^+^ and CXCR3^+^ and increase of CD16^+^ CD56^bright^ NK cells in untreated HIV-seropositive blood donors compared to healthy controls (Fig. 1B). These alterations were partially reversed in patients on HAART with suppressed viral loads for more than one year although the decrease of frequencies of CD16^+^CD56^bright^ NK cells in treated subjects compared to untreated patients did not reach statistical significance. Notably, the relative loss of CXCR3^+^CD56^bright^ cells was not reversed in HIV-infected patients after treatment. Analysis of HIV-patients, which had been treated for less than one year, still exhibited loss of CCR7-expressing CD56^bright^ NK cells despite suppression of viral load below detection limits (data not shown). This suggests that reversing the impact of HIV-infection on CD56^bright^ NK cell phenotype requires time.

Due to their involvement in lymphocyte homing CCR7 and CD62L are frequently co-expressed on T cells [23]. Furthermore, the presence of CD62L on NK cells has been associated with a polyfunctional cell profile and CD62L is highly expressed on CD56^bright^ NK cells [24]. Notably, and in contrast to our observations of decreased frequencies of CCR7^+^CD56^bright^ NK cells, no significant alterations were found in terms of numbers of CD62L-expressing CD56^bright^ NK cells in HIV-infection (Fig. 1B). There was no correlation between the rise of CCR7^−^CD56^bright^ NK cells and percentages of CD16-expressing cells and only a modest correlation between frequencies of CCR7 and CD62L expressing CD56^bright^ NK cells (Fig. 1C). We also assessed possible relationships between the decreased percentages of CXCR3^+^CD56^bright^ NK cells with either percentages of CCR7^+^CD56^bright^ or CD16^+^CD56^bright^ NK cells but were unable to find significant correlations (Figure S1A) and the percentages of CXCR3^+^ cells did not vary in CCR7^+^ compared to CCR7^−^CD56^bright^ NK cells (Figure S1B).

Our cohort of untreated HIV-infected patients comprised elite viral controllers as well as non-controllers with rapid disease progression. This prompted us to analyze whether HIV viral loads were associated with the observed decrease of CCR7-expressing CD56^bright^ NK cells. Indeed, we found highly significant inverse correlation between frequencies of CCR7^+^CD56^bright^ NK cells and HIV-RNA copies/ml in these untreated patients (Fig. 1D). In addition, there was a weaker yet significant correlation between percentages of CCR7^+^CD56^bright^ or CXCR3^+^CD56^bright^ NK cells with CD4^+^ T cell counts (Fig. 1D). No correlations with viral copy numbers and CD4^+^ T cell counts were found for CD16- or CD62L-expressing CD56^bright^ NK cells. We thus demonstrate a direct correlation of loss of CCR7-expressing but not CD16- or CD62L-expressing CD56^bright^ NK cells with these clinical disease parameters.

**Figure 2:**
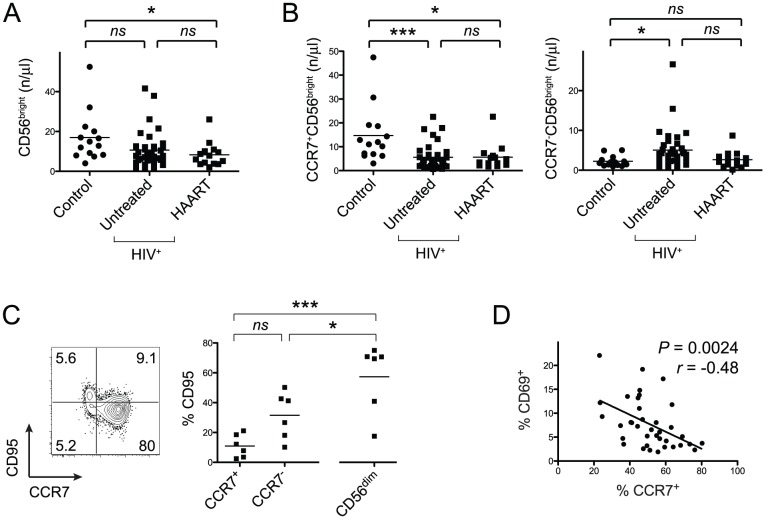
Relative and absolute loss of CCR7^+^CD56^bright^ NK cells is not attributable to apoptosis. (A) Absolute cell numbers of CD56^bright^ NK cells are depicted. Horizontal bars indicate means. (B) Absolute cell numbers of either CCR7^+^ or CCR7^−^CD56^bright^ NK cells are shown. (C) Representative flow cytometry data of CD95 on gated CD56^bright^ NK cells and respective summary data derived from untreated HIV-patients. Numbers in flow cytometry plots indicate frequencies of quadrants and horizontal bars in dot plot indicate mean values. (D) Pearson’s correlation analysis between frequencies of CCR7- and CD69-expressing CD56^bright^ NK cells. *, *P*<0.05; ***, *P*<0.001; *NS* – not significant.

The absolute numbers of CD56^bright^ NK cells in untreated HIV-infected patients compared to healthy controls were not substantially decreased (Fig. 2A). However, the relative loss of CCR7-expressing CD56^bright^ NK cells was also reflected in their absolute cell numbers and we also detected increased absolute counts of CCR7^−^CD56^bright^ NK cells in untreated HIV-seropositive subjects (Fig. 2B).

We next tested the hypothesis whether the relative and absolute decrease of CCR7^+^C56^bright^ NK cells in chronic HIV-infection was due to their increased susceptibility of apoptosis. To address this question we measured expression of Fas (CD95) on NK cell subsets in freshly isolated PBMC samples. There were no statistically significant differences when comparing CCR7^-^CD56^bright^, CCR7^+^CD56^bright^ and CD56^dim^CD16^+^ NK cells using a One-Way ANOVA followed by a Tukey post-test (Fig. 2C). However, when we tested the hypothesis whether CCR7^-^CD56^bright^ and CCR7^+^CD56^bright^ NK cells differed in the percentages of CD95-expressing cells, we found higher frequencies among CCR7^-^CD56^bright^ NK cells (*P*<0.016, t test). Highest frequencies were found on CD56^dim^CD16^+^ NK cells (Fig. 2C). The frequency of CD95-expressing CCR7^+^CD56^bright^ NK cells was slightly elevated in untreated HIV-infected patients compared to uninfected control subjects. (Figure S2A) and we observed a non-significant negative association between percentages of CD95^+^ and CCR7^+^CD56^bright^ NK cells (Figure S2B). We also studied the frequencies of CD56^bright^ NK cells expressing the TNF receptor type II (CD120b) and found that CD56^bright^ NK cells from untreated HIV-positive subjects exhibited higher percentages of CD120^+^ cells (Figure S2C). Nonetheless, frequencies of CD120b^+^ cells ranged at relatively low levels with an average percentage of lower than 10%. Unlike CD95, relative numbers of CD120^+^ cells did not vary significantly in CCR7^+^ compared to CCR7^−^CD56^bright^ cells (Figure S2D). Highest frequencies of CD120^+^ cells were found within the CD56^dim^CD16^+^ NK cell subpopulation. In addition, there was no detectable expression of TNF-related apoptosis-inducing ligand receptor 2 (TRAIL-R2) on NK cells in freshly isolated PBMCs from HIV-patients (data not shown) and a previous study suggested that NK cells are resistant to TRAIL-mediated apoptosis [25]. We found no significant intracellular expression of Caspase-3 or Bcl-2 in CCR7^−^ and CCR7^+^CD56^bright^ NK cells *ex vivo* (data not shown) indicating low or undetectable apoptosis levels within the CD56^bright^ NK cell subset in freshly isolated PBMCs.

Cytokine-induced activation of NK cells can lead to down-regulation of CCR7 after several days [17]. Thus, a plausible explanation for decreased numbers of CCR7^+^CD56^bright^ NK cells despite stable numbers of total CD56^bright^ NK cells could be an overall activated state of the immune system in HIV-seropositive subjects. Indeed, we detected a moderate yet significant negative correlation between frequencies of CD56^bright^ NK cells expressing the activation marker CD69 and CCR7^+^CD56^bright^ NK cells (Fig. 2D). This suggests that immune activation is a correlate for the alterations of the CD56^bright^ NK cells subset.

**Figure 3:**
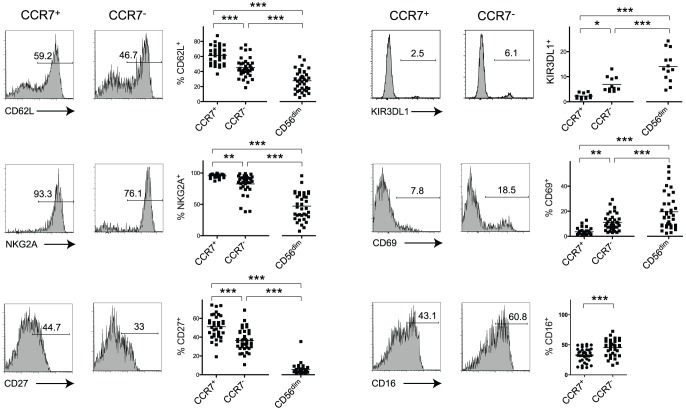
CCR7^−^CD56^bright^ NK cells exhibit phenotypic features of CD56^dim^CD16^+^ cells. Representative expression data of CD62L, NKG2A, CD27, KIR3DL1, CD69 and CD16 on gated CCR7^+^ or CCR7^−^ CD56^bright^ cells and respective summary data including CD56^dim^CD16^+^ NK cells, from untreated HIV-seropositive individuals. Numbers represent percentages of gated events and horizontal bars in dot plots indicate mean values. *, *P*<0.05; **, *P*<0.01; ***, *P*<0.001.

We next sought to answer the question whether loss of CCR7 on CD56^bright^ NK cells was associated with an altered phenotype. We identified a decrease of CD62L-, NKG2A- and CD27-expressing cells among CCR7^−^CD56^bright^ NK cells compared to CCR7^+^CD56^bright^ NK cells in untreated HIV-seropositive individuals (Fig. 3). A further decrease of percentages of CD62L^+^, NKG2A^+^ and CD27^+^ cells could be seen among CD56^dim^CD16^+^NK cells compared to CCR7^+^CD56^bright^ NK cells or compared to CCR7^−^CD56^bright^ NK cells (Fig. 3).

NK cell differentiation was previously shown to be accompanied by an increase in KIR-expressing cells [5], [9], [18]. The expression of KIR3DL1 (CD158e) was highest on CD56^dim^CD16^+^ NK cells but CCR7^−^CD56^bright^ cells had an increase of KIR3DL1-expressing cells compared to CCR7^+^ cells (Fig. 3). We also found considerable increase of cells expressing CD69 among CCR7^−^CD56^bright^ NK cells in comparison with their CCR7^+^ counterpart, which was again exceeded by the levels of CD56^dim^CD16^+^ NK cells suggesting higher activation states in these populations (Fig. 3). Furthermore, moderately higher percentages of CD16^+^ cells were found among CCR7^−^CD56^bright^ NK cells. Because we were unable to identify a correlation between frequencies of CCR7^+^CD56^bright^ and CD16^+^CD56^bright^ NK cells (Fig. 1C) we addressed the question whether frequencies of CCR7^+^ cells varied between CD16^+^ and CD16^−^CD56^bright^ NK cells. CD16^+^CD56^bright^ NK cells exhibited a small but statistically significant decrease of CCR7-expressing cells (Figure S3A). Altogether, our data indicate that HIV-associated CCR7^−^CD56^bright^ NK cells display an ‘intermediate’ phenotype sharing properties of CD56^bright^ as well as CD56^dim^CD16^+^ NK cells.

Overall, CCR7^−^CD56^bright^ NK cells are only present at low frequencies in uninfected individuals. We hypothesized that the intermediate phenotype of CCR7^−^CD56^bright^ NK cells was not exclusively induced by chronic HIV infection but might also be present at lower frequencies in healthy control subjects. We were indeed able to identify a number of control individuals and HIV-patients on HAART with moderate numbers of CCR7^−^CD56^bright^ NK cells. Overall, there were similar trends in terms of phenotypic differences between CCR7^−^ and CCR7^+^CD56^bright^ NK cells (Figure S3B, C). These findings therefore imply that CCR7^−^CD56^bright^ NK cells do occur at low frequencies in HIV-seronegative subjects and HAART-treated individuals and that these cells display similar phenotypes to the ones observed in CCR7^−^CD56^bright^ cells in viremic HIV-patients.

**Figure 4:**
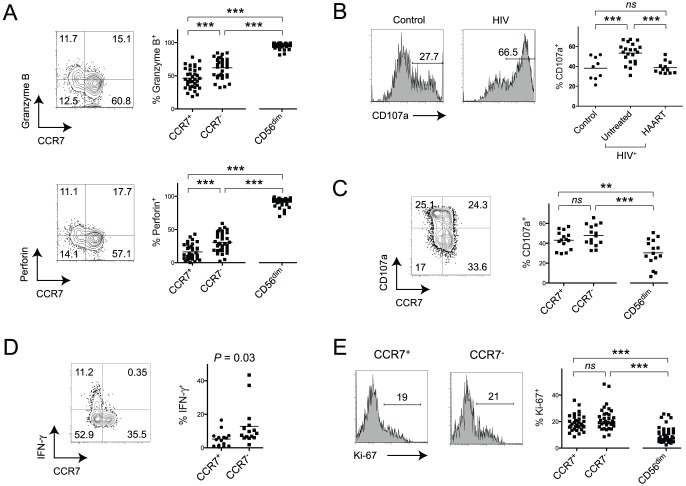
Functional differences between CCR7 ^−^ **and CCR7^+^CD56^bright^ NK cells from HIV-infected donors indicate high activation states.** (A) Representative flow cytometry plots of granzyme B and perforin expression on gated CD56^bright^ NK cells and summary data including CD56^dim^CD16^+^ cells, from untreated HIV-infected subjects. Horizontal bars in dot plots show mean values. Numbers in corners represent percentage of quadrant. (B) Representative histograms and summary data of CD107a degranulation in CD56^bright^ cells from uninfected controls, untreated and HAART-treated HIV-patients. Data was generated using sorted NK cells stimulated with IL-12, IL-15 and K452 cells. (C) Representative flow cytometry plot of CD107a degranulation on gated CD56^bright^ NK cells and summary data of degranulation in CCR7^+^CD56^bright^, CCR7^−^CD56^bright^ and CD56^dim^ NK cells from untreated HIV-infected subjects is shown. Data was generated using whole PBMCs stimulated with IL-12, IL-15 and K562 cells. Numbers in corners represent percentage of quadrant. (D) Spontaneous expression of IFN-γ in medium-only treated NK cell subsets is shown in a representative flow cytometry plot and a summary data graph. Data from untreated HIV-positive patients is shown and numbers in corners indicate percentages of quadrants. (E) Representative Ki-67-expression data and summary data on gated CCR7^+^ or CCR7^−^ CD56^bright^ cells and respective summary data including CD56^dim^CD16^+^ NK cells from untreated HIV-seropositive subjects. Numbers in flow cytometry histogram plots indicate percentage of gated events. ***, *P*<0.001; *NS* – not significant.

We next addressed the question whether CCR7^−^CD56^bright^ NK cells display functional characteristics of CD56^dim^CD16^+^ NK cells. Percentages of granzyme B as well as perforin producing cells among CCR7^−^CD56^bright^ NK cells were significantly augmented in comparison with CCR7^+^CD56^bright^ NK cells in untreated HIV-seropositive subjects (Fig. 4A). Importantly, numbers of granzyme B^+^ and perforin^+^ of CCR7^−^CD56^bright^ NK cells were again between the levels of the CCR7^+^CD56^bright^ NK cell subset and CD56^dim^CD16^+^ NK cells (Fig. 4A). Similar trends were observed in uninfected subjects (Figure S3D).

We next assessed the ability of total CD56^bright^ NK cells to degranulate, which was shown to be closely related to the cytotoxic activity of NK cells [26], [27]. Treatment of NK cells with IL-12 and IL-15 only induced weak degranulation of CD56^bright^ NK cells and the average percentage of CD107a-expression was 6.6% (data not shown). We thus treated sorted NK cells with IL-12, IL-15 and K562 cells to achieve robust activation of NK cells and to be able to measure cytokine-production and degranulation at the same time. Notably, there was a significant increase of CD107a-expressing CD56^bright^ NK cells from untreated HIV-infected patients compared to control subjects, which was reversed in treated patients (Fig. 4B). Next, we addressed the question whether higher numbers of degranulating cells could be found among CCR7^−^CD56^bright^ NK cells compared to CCR7^+^CD56^bright^ cells in untreated HIV-infected subjects. There was only a non-significant, minor increase of CD107a-expressing cells among CCR7^−^CD56^bright^ NK cells (Fig. 4C). We also evaluated the ability of CD56^bright^ NK cells to produce cytokines. Notably, we found increased numbers of cells expressing IFN-γ in CCR7^−^CD56^bright^ NK cells in medium-treated PBMCs without further stimulation (Fig. 4D), which corroborates our hypothesis that these cells display a more activated phenotype *ex vivo*. Upon stimulation we observed a slight increase of IFN-γ-expressing CD56^bright^ NK cells in untreated HIV-seropositive individuals compared to uninfected subjects as well as a non-significant increase of TNF-expressing cells (Figure S4A).

We next sought to answer the question whether occurrence of CCR7^−^CD56^bright^ NK cells in untreated, chronic HIV-infection was associated with increased *in vivo* proliferation. Higher frequencies of Ki-67^+^ cells were found among CD56^bright^ NK cells in comparison with the CD56^dim^CD16^+^ subset (Fig. 4E). However, we were not able to detect a significant difference between CCR7^−^ and CCR7^+^CD56^bright^ NK cells (Fig. 4E). In accordance with a previous study [13], there were higher numbers of Ki-67^+^ NK cells in untreated HIV-infected patients compared to the uninfected control cohort in every NK cell subset we tested (Figure S4B).

Since our data suggested that CCR7^−^CD56^bright^ NK cells could represent an intermediate NK cell subset in the context of a possible differentiation pathway from CD56^bright^ to CD56^dim^CD16^+^ NK cells, we wondered whether we could reproduce some of our *ex vivo* observations in an *in vitro* culture model. To test this hypothesis, we sorted CCR7^+^CD56^bright^ NK cells from uninfected blood donors (Figure S5A) and cultured these cells either in the presence or absence of 100 U/ml IL-2, 25 ng/ml IL-12 and 25 ng/ml IL-15. Culturing CCR7^+^CD56^bright^ NK cells in medium alone for five days was not sufficient to induce significant loss of CCR7 expression (Figure S5B). However, cytokine-treated CCR7^+^CD56^bright^ NK cells gradually exhibited an increasing loss of CCR7 by days 3 and 5 (Figure S5B). Furthermore, treatment of cells with cytokines induced the expression of granzyme B and perforin whereas medium alone was not sufficient to substantially up-regulate these proteins (Figure S5C). However, it should be emphasized that the down-regulation of CCR7 was only modest and we did not observe a significant downregulation of CD56. These results thus indicate that cytokine treatment of CCR7^+^CD56^bright^ NK cells can partially produce a phenotype similar to the altered profile of CD56^bright^ NK cells in chronic HIV-infection.

## Discussion

A number of previous studies suggested that CD56^bright^ NK cells are less differentiated progenitors of CD56^dim^CD16^+^ NK cells [5], [9], [15]–[18]. In support of this model, intermediate subsets of NK cells have been identified. Within the CD56^dim^ NK cells, a subset of CD94^high^-expressing cells was shown to have an intermediate profile as shown by expression levels of CD62L, CD2, KIRs, granzyme B, perforin and functional markers, which ranged between the CD56^bright^ and the CD94^low^CD56^dim^ NK cell subset [5]. Furthermore, a recent study proposed a CD16-expressing subset within the CD56^bright^ NK cells to be an intermediate stage of NK cell differentiation indicated by their phenotypic and functional features [19]. However, not much is known about the impact of chronic HIV-infection on CD56^bright^ NK cells or putative alterations of intermediate NK cell populations.

Here, we provide an extensive phenotypic analysis of the alterations of CD56^bright^ NK cells in chronic HIV infection. We demonstrate that untreated HIV-infection is associated with increased numbers of CD56^bright^ NK cells expressing CD16 and a loss of CXCR3-expressing cells. We report a substantial relative and absolute increase of CCR7^−^CD56^bright^ NK cells and conversely diminished numbers of CCR7^+^CD56^bright^ NK cells in viremic HIV-1 infection. However, whereas the percentage of CCR7^+^CD56^bright^ NK cells was almost restored to normal levels after prolonged anti-retroviral treatment, absolute numbers of CCR7^+^CD56^bright^ cells remained at lower levels in treated patients. We also demonstrate overall lower absolute counts of CD56^bright^ NK cells in our cohort of treated HIV-patients compared to uninfected subjects. Thus, the unrestored absolute numbers of CCR7^+^CD56^bright^ NK cells in the cohort of treated HIV-patients could represent a reflection of the low absolute CD56^bright^ NK cell counts.

The relative increase of CD16^+^CD56^bright^ NK cells could suggest an impact of HIV on a previously characterized intermediate NK cell subpopulation [19]. However, this increase did neither correlate with viral load or CD4^+^ T cell counts, nor did we find correlations with diminished frequencies of CCR7^+^CD56^bright^ NK cells. In addition, although there was a positive correlation between relative numbers of CXCR3^+^CD56^bright^ NK cells and absolute T cell counts, there was no correlation with viral load or with CCR7^+^CD56^bright^ NK cells. Our data therefore suggest that the loss of CCR7-expressing CD56^bright^ NK cells is distinct from up-regulation of CD16 or loss of CXCR3^+^CD56^bright^ cells.

Furthermore, we demonstrated that the CCR7^−^CD56^bright^ subpopulation displays a number of similarities with CD56^dim^CD16^+^ NK cells as shown by increased frequencies of cells expressing granzyme B, perforin, KIR, CD16 and decreased numbers of cells expressing CD62L, NKG2A and CD27. Despite elevated cell numbers expressing granzyme B and perforin, the CCR7^−^CD56^bright^ NK cell subset exhibited similar levels of degranulation compared to CCR7^+^CD56^bright^ cells but higher spontaneous IFN-γ production. Increased cytolytic properties of CCR7^−^CD56^bright^ NK cells were not accompanied by significantly enhanced proliferative activity as monitored by intracellular Ki67 staining. Altogether, the loss of CCR7 on CD56^bright^ NK cells marks a phenotypic shift of CD56^bright^ NK cells towards a CD56^dim^CD16^+^ NK cell-like phenotype, which strongly correlates with clinical parameters of HIV-associated immune disease. Importantly, CCR7^−^CD56^bright^ NK cells occur only at relatively low frequencies in uninfected subjects and exhibit comparable phenotypic properties to the CCR7^−^CD56^bright^ NK cell population in HIV-infected patients.

The loss of CCR7 could imply a loss of migratory capacity to lymph nodes. Interestingly, a previous study in rhesus macaques reported a loss of CCR7-expressing but not CD62L-expressing CD56^+^ NK cells after SIV-infection [28]. Similar to our data, Reeves *et al*. reported an increase of granzyme B- and perforin-expression and higher activation states in CD56^+^ NK cells. In SIV-infected macaques, the loss of CCR7 on NK cells was accompanied by an increase of gut-homing receptor α4ß7-expression implying trafficking of NK cells into gut mucosal tissues [29].

Our observations could potentially be explained by an up-regulation of CD56 on activated CD56^dim^CD16^+^ NK cells. However, the scarce expression of KIRs (Fig. 3) and the absence of CD57 as well as the high expression profiles of CD94 (data not shown) on CCR7^-^CD56^bright^ NK cells make this possibility less plausible. In addition, apoptosis had been suggested to play a critical role in the overall decrease of absolute numbers of NK cells [13]. Our findings however indicate overall relatively low numbers of CD95^+^ and CD120b^+^ cells among CD56^bright^ NK cells. In addition, there was a two-fold increase of percentages of CD95^+^ cells among the expanded CCR7^-^CD56^bright^ NK cell subset compared to the decreased CCR7^+^CD56^bright^ cell subset. Thus, even though we cannot conclusively rule out a potential contribution of apoptosis to the loss of CCR7^+^CD56^bright^ NK cells our results indicate that it is unlikely that apoptosis is the defining cause for the selective depletion of the CCR7^+^CD56^bright^ subset. These data are in accordance with previous findings that Fas-mediated apoptosis in viremic HIV-1 patients is more frequently found within the more differentiated CD56^dim^CD16^+^ NK cell population [13].

One of the hallmarks of chronic HIV-infection is systemic immune activation of the host [30]. The finding that CD69-expression is substantially increased on the CCR7^-^CD56^bright^ population supports a dominant role for immune activation in the observed alteration of this NK cell phenotype in chronic HIV-1 infection. Similar observations have also been described on CD56^dim^CD16^+^ and CD56^neg^CD16^+^ NK cells in HIV-1 infection [9], [12]. In support of this notion, we identified a negative correlation between percentages of CD69-expressing CD56^bright^ cells and CCR7^+^CD56^bright^ NK cells. This finding indicates that activation of NK cells is a correlate for the relative loss of the CCR7^+^CD56^bright^ NK cell subpopulation. Human NK cells express various cytokine receptors [1] and a previous study showed that stimulation with either IL-2 or IL-12 was enough to induce loss of CCR7 on CD56^bright^ cells and acquisition of CD56^dim^CD16^+^ cell resembling characteristics, such as granzyme B expression clearly suggesting that cytokines can induce NK cell differentiation [17]. Notably, we were able to generate similar data when we cultured and cytokine-activated highly purified CCR7^+^CD56^bright^ NK cells instead of using bulk CD56^bright^ NK cells. Thus, it is tempting to speculate that CCR7^+^CD56^bright^ NK cells could represent a less differentiated NK cell subset. However, in our *in vitro* system we were unable to demonstrate a full transformation of CCR7^+^CD56^bright^ cells into CD56^dim^ NK cells, which is in accordance with a previous study [16], suggesting the involvement of additional factors in the process of NK cell differentiation, such as interaction with tissue fibroblasts. Further studies in appropriate animal models such as humanized mice or nonhuman primates could be helpful to better define the precise ontogeny of both, CCR7^+^ and CCR7^−^CD56^bright^ NK cells.

Importantly, the phenotypic and functional skewing of CD56^bright^ NK cells, which we report in this manuscript, may not be limited to chronic HIV-infection. A number of viruses are able to evade a complete eradication by the host immune surveillance to establish a chronic, life-long infection in humans [31]. Similarly to HIV, hepatitis B virus (HBV) and hepatitis C virus (HCV) are known to undergo continuous cycles of replication during chronic infection, thus providing constant antigenic stimulation of host lymphocytes, which could also induce NK cell activation [32]. It will thus be interesting to see if and how chronic viral infections can impact NK cell differentiation stages in humans although it should be noted that the degree of systemic, chronic immune activation in HIV pathogenesis seems to exceed other known chronic viral infections in humans.

In summary, we provide a thorough characterization of the changes within the CD56^bright^ NK cell subset in chronic HIV infection, which is reflected in a shift towards CD56^dim^CD16^+^ NK cells thus providing a novel aspect of HIV-associated alterations of the NK cell compartment.

## Supporting Information

Figure S1 **Alterations of CXCR3 expression on CD56^bright^ NK cells do not correlate with alterations of CCR7 expression in chronic HIV infection.** (A) Pearson’s correlation analyses between frequencies of CXCR3^+^CD56^bright^ NK cells and CCR7^+^CD56^bright^ or CD16^+^CD56^bright^ NK cells in untreated HIV-seropositive patients are shown. (B) The frequencies of CXCR3^+^ cells in CCR7^+^, CCR7^−^CD56^bright^ or CD56^dim^ NK cells derived from untreated HIV-seropositive individuals are shown. Horizontal bars indicate means. *, *P*<0.05; **, *P*<0.01; ***, *P*<0.001; *NS* – not significant.
(EPS)

Figure S2 **CD95 and CD120b are up-regulated on NK cell subpopulations in chronic HIV-infection.** (A) Summary data of frequencies of CD95-expressing CCR7^+^CD56^bright^ NK cells is shown in uninfected, treated and untreated HIV-positive individuals. (B) Pearson’s correlation analysis between frequencies of CD95^+^CD56^bright^ NK cells and CCR7^+^CD56^bright^ NK cells in treated and untreated HIV-seropositive patients. (C) Summary data of percentages of CD120b^+^CD56^bright^ NK cells is shown. Horizontal bars represent means. (D) The frequencies of CD120b^+^ cells in CCR7^+^, CCR7^−^CD56^bright^ or CD56^dim^ NK cells derived from untreated HIV-seropositive individuals are shown. Horizontal bars indicate means. ***, *P*<0.001.
(EPS)

Figure S3 **Phenotypical differences between CCR7^−^ and CCR7^+^CD56^bright^ NK cells observed in healthy controls and HAART-treated HIV-1 infected patients.** (A) The frequencies of CCR7^+^ cells in CD16^+^ and CD16^−^CD56^bright^ NK cells are shown. Horizontal lines represent means. (B) Summary data showing percentages of CD62L-, CD69- and CD16-expressing cells of either CCR7^+^ or CCR7^−^CD56^bright^ NK cells or CD56^dim^CD16^+^ NK cells. Horizontal lines depict means. (C) The percentage of cells displaying NKG2A and CD27 of CCR7^+^ or CCR7^−^CD56^bright^ NK cells or CD56^dim^CD16^+^ NK cells is shown in HIV-seronegative individuals (n = 4). (D) The frequencies of cells expressing intracellular granzyme B and perforin of CCR7^+^ or CCR7^−^CD56^bright^ NK cells or CD56^dim^CD16^+^ NK cells are shown in uninfected control subjects (n = 3). **, *P*<0.01; ***, *P*<0.001.
(EPS)

Figure S4 **Functional alterations indicate a higher activation status of NK cells in untreated HIV-seropositive individuals.** (A) Sorted NK cells were stimulated with IL-12, IL-15 and K562 cells and the percentages of cells expressing IFN-γ and TNF-α were measured in CD56^bright^ NK cells derived from untreated HIV-positive and uninfected control donors. Horizontal bars represent means. (B) The frequencies of Ki-67-expressing cells in CCR7^+^CD56^bright^, total CD56^bright^ and CD56^dim^CD16^+^ NK cells are shown. Untreated HIV-seropositive and uninfected control donors were compared. Horizontal bars represent means.
(EPS)

Figure S5 **Cytokine-treatment of CCR7^+^CD56^bright^ cells can induce NK cell differentiation.** (A) Highly purified CCR7^+^CD56^bright^ NK cells were obtained from uninfected blood donors by cell sorting. Numbers in representative flow cytometry plots indicate frequencies of gated events. (B) Expression of CCR7 is shown at day 1, 3 and day 5 of cell culture. Numbers in corners indicate the percentages of quadrants. Data is representative for two independent experiments with similar results. (C) Expression of granzyme B and perforin is shown at day 0, 3 and 5 of culturing CCR7^+^CD56^bright^ in medium or in the presence of cytokines. Representative data of two independent experiments is shown.
(EPS)

Table S1 **Demographic data of HIV-infected study subjects.** Data is shown for each HIV-seropositive study participant.
(DOCX)
