# Supplementary material for: Loss of CCR7 Expression on CD56bright NK Cells Is Associated with a CD56dimCD16+ NK Cell-Like Phenotype and Correlates with HIV Viral Load
Source: PLoS One. 2012 Sep 19;7(9):e44820. doi: 10.1371/journal.pone.0044820 (PMC3447005; doi:10.1371/journal.pone.0044820)
Supplement: Table S1 — Demographic data of HIV-infected study subjects. Data is shown for each HIV-seropositive study participant. (DOCX) [file pone.0044820.s006.docx]

**Supplementary table 1.** Demographic data of HIV-infected study subjects.

| **Patient ID** | **Treatment** | **Sex** | **Age** | **CD4 T cell count (n/ml)** | **Viral load (copies/ml)** | **CD4/CD8 T cell ratio** |
| --- | --- | --- | --- | --- | --- | --- |
| UV184 | HAART | female | 39 | 538 | < 47 | 0.74 |
| UV126 | HAART | male | 49 | 386 | 33 | 0.41 |
| UV017 | HAART | female | 44 | 330 | < 47 | 0.56 |
| UV018 | HAART | male | 47 | 293 | 33 | 0.47 |
| UV008 | HAART | female | 32 | 732 | 55 | 1.37 |
| UV042 | HAART | male | 64 | 414 | < 47 | 0.9 |
| UV196 | HAART | male | 47 | 151 | 33 | 0.32 |
| UV094 | HAART | male | 47 | 809 | < 47 | 0.81 |
| UV193 | HAART | female | 48 | 419 | < 47 | 0.5 |
| UV022 | HAART | male | 34 | 557 | < 47 | 0.59 |
| UV 076 | HAART | male | 68 | 441 | < 47,0 | 0.83 |
| UV 138 | HAART | male | 41 | 490 | < 47,0 | 0.6 |
| UV 225 | HAART | male | 38 | 410 | < 47,0 | 0.92 |
| UV 049 | HAART | Male | 41 | 950 | < 47.0 | 0.62 |
| UV022 | HAART | male | 26 | 285 | 49 | 0.31 |
| UV173 | Untreated | male | 34 | 1355 | 33 | 1 |
| UV147 | Untreated | female | 43 | 845 | 33 | 0.87 |
| UV162 | Untreated | male | 57 | 1281 | 33 | 1.63 |
| UV146 | Untreated | male | 43 | 736 | 49 | 1.27 |
| UV193 | Untreated | male | 39 | 592 | 110 | 0.59 |
| UV169 | Untreated | female | 32 | 754 | 621 | 0.6 |
| UV096 | Untreated | male | 40 | 363 | 881 | 0.31 |
| AA062 | Untreated | male | 43 | 476 | 1699 | 0.6 |
| UV180 | Untreated | male | 73 | 393 | 1710 | 0.38 |
| UV163 | Untreated | female | 22 | 514 | 1750 | 0.6 |
| UV171 | Untreated | male | 42 | 748 | 1910 | 0.6 |
| UV178 | Untreated | male | 48 | 382 | 2280 | 0.21 |
| UV090 | Untreated | female | 32 | 630 | 2870 | 0.48 |
| UV189 | Untreated | female | 49 | 510 | 2990 | 0.51 |
| AA069 | Untreated | male |  | 151 | 5030 | 0.3 |
| UV191 | Untreated | male | 24 | 400 | 6510 | 0.51 |
| UV187 | Untreated | female | 24 | 351 | 10900 | 0.29 |
| UV129 | Untreated | male | 47 | 353 | 11300 | 0.34 |
| UV002 | Untreated | female | 26 | 286 | 11400 | 0.41 |
| UV046 | Untreated | female | 31 | 338 | 11500 | 0.63 |
| UV190 | Untreated | male | 26 | 357 | 11500 | 0.75 |
| UV181 | Untreated | male | 61 | 217 | 13600 | 0.42 |
| UV085 | Untreated | female | 69 | 180 | 13800 | 0.15 |
| UV167 | Untreated | male | 28 | 337 | 15500 | 0.29 |
| UV182 | Untreated | male | 42 | 332 | 21000 | 0.26 |
| UV114 | Untreated | male | 48 | 264 | 26500 | 0.19 |
| UV151 | Untreated | male | 45 | 467 | 28300 | 0.21 |
| AA066 | Untreated | male | 66 | 581 | 29900 | 0.42 |
| UV160 | Untreated | female | 41 | 283 | 41200 | 0.13 |
| UV161 | Untreated | male | 20 | 442 | 43400 | 0.51 |
| UV174 | Untreated | male | 44 | 219 | 52600 | 0.16 |
| UV179 | Untreated | male | 28 | 480 | 60200 | 0.48 |
| UV175 | Untreated | male | 30 | 356 | 70900 | 0.35 |
| UV168 | Untreated | male | 64 | 200 | 89300 | 0.23 |
| UV036 | Untreated | female | 31 | 770 | 93300 | 0.76 |
| UV188 | Untreated | male | 48 | 291 | 120000 | 0.48 |
| UV185 | Untreated | female | 31 | 304 | 158000 | 0.21 |
| UV105 | Untreated | male | 37 | 199 | 1370000 | 0.27 |
